# Cross-talks between perivascular adipose tissue and neighbors: multifaceted nature of nereids

**DOI:** 10.3389/fphar.2024.1442086

**Published:** 2024-08-02

**Authors:** Yujuan Li, Zhang Chen, Ying Xiao, Xinzhi Li

**Affiliations:** ^1^ School of Pharmacy, Faculty of Medicine, Macau University of Science and Technology, Macau, China; ^2^ (R & D Center) Laboratory for Drug Discovery from Natural Resource, Macau University of Science and Technology, Macau, China; ^3^ State Key Laboratory of Quality Research in Chinese Medicine, Macau University of Science and Technology, Macau, China; ^4^ Faculty of Medicine, Macau University of Science and Technology, Macau, China

**Keywords:** perivascular adipose tissue, secretome, intercellular communication, obesity, cardiovascular disease

## Abstract

Perivascular adipose tissue (PVAT) is a unique fat depot surrounding blood vessels and plays a vital role in the progression of vascular remodeling and dysfunction. PVAT exhibits remarkable differences in structure, phenotype, origin, and secretome across anatomical locations. The proximity of PVAT to neighboring vascular beds favors a niche for bidirectional communication between adipocytes and vascular smooth muscle cells, endothelial cells, and immune cells. In this review, we update our understanding of PVAT’s regional differences and provide a comprehensive exploration of how these differences impact cross-talks between PVAT and the vascular wall. Different PVAT depots show different degrees of vasoprotective function and resilience to pathological changes such as obesity and vasculopathies, shaping multifaceted interactions between PVAT depots and adjacent vasculatures. The depot-specific resilience may lead to innovative strategies to manage cardiometabolic disorders.

## 1 Introduction

Cardiovascular disease is the leading cause of death worldwide. Obesity and type 2 diabetes mellitus are two predominant risk factors of cardiometabolic diseases, including arterial stiffness, atherosclerosis, and aortic aneurysm, which are all featured by dyslipidemia and chronic vascular inflammation (Rocha and Libby, 2009; Ortega et al., 2016). PVAT around the blood vessels has been considered as an important fat depot for understanding correlation between dyslipidemia and cardiovascular disease. In addition to mechanical support, PVAT functions as an endocrine organ that controls vascular tone by secreting adipokines (Adachi et al., 2022; Huang et al., 2023). Under normal physiological condition, PVAT can secret protective adipokines which exert anti-inflammatory and vasodilatory effects (Fésüs et al., 2007; Adachi et al., 2022). However, in obesity, PVAT undergoes dysfunction and exhibits an abnormality in secretome, leading to vascular inflammation in a so-called “outside-in” manner, where the pathological cascade is advanced from outside adipose tissue to inside endothelium (Chatterjee et al., 2009; Police et al., 2009; Bailey-Downs et al., 2013). Meanwhile, vasculopathies trigger an “inside-out” paradigm, where the impaired vascular wall initiates local oxidative stress and inflammation, subsequently impacting PVAT function (Song et al., 2012). The vicious bidirectional interaction between PVAT and blood vessels contributes to develop a range of cardiometabolic disorders (Lohmann et al., 2009).

Different vasculatures are surrounded by diverse PVAT depots. Aortas generally harbor larger amounts of beige or brown PVATs that tend to produce heat and provide energy through burning triglyceride, while smaller arteries are wrapped by white PVATs, which take up excessive free fatty acids and store energy in the form of lipid droplet. The PVAT enveloping the thoracic aorta from the aortic arch to diaphragm exhibits different histology, phenotype and secretome from the PVAT attached to the abdominal aorta (Huang et al., 2023). In addition, the coronary artery is enveloped in a unique PVAT, which differs from the PVAT surrounding the mesenteric artery (de Jong et al., 2015; Gaborit et al., 2015). It is widely recognized that the onset of atherosclerosis and aortic aneurysms varies significantly depending on the location of the blood vessels. This observation suggests that the unique characteristics of PVAT in various vascular beds could undoubtedly shape the distribution and severity of vasculopathy. As in the captivating story of the Nereids from Greek mythology, the depot-specific features of PVAT resemble the diverse attributes possessed by each Nereid. Just as these sea nymphs were known for their distinct characteristics and roles, PVAT in different vascular regions exhibits unique properties that contribute to the multifaceted nature of this specialized adipose tissue.

In our review published in 2021 (Li et al., 2021), we summarized the heterogeneity of PVATs in morphology, origin, and secretome. In addition to updating our understanding of the biological differences among PVAT depots, the current review primarily focuses on cross-talks between PVAT depots and nearby arteries, particularly highlighting the bi-directional interaction in response to obesity and vasculopathies. We continue to use the same nomenclature to indicate the specific PVAT throughout the present review as we did previously (Li et al., 2021), where a common name, “PVAT,” follows a lowercase letter, indicating where it is localized. For example, pericoronary adipose tissue is termed as “cPVAT,” thoracic periaortic adipose tissue is abbreviated as “tPVAT,” and abdominal periaortic adipose tissue is named as “aPVAT.” Adipose tissue surrounding the mesenteric artery is called “mPVAT.”

## 2 Biological differences between PVAT depots: anatomy, phenotype, and origin

### 2.1 Anatomic and phenotypical differences of PVAT depots

PVAT depots exhibit distinct phenotypes at different anatomical location, and this distinction is primarily determined by its tissue composition, readily reflected by tissue color, namely, white, brown, or beige (also known as brite, brown-in-white) adipose tissue. In rodents, thoracic aortic PVAT (tPVAT) consist primarily of brown adipocytes with multilocular lipid droplets and higher mitochondrial density (Tran et al., 2018). The tPVAT expressed high levels of brown adipocyte markers, such as uncoupling protein-1(*Ucp1*), PRD1-BF1-RIZ1 homologous domain containing 16 (*Prdm16*), peroxisome proliferator-activated receptor gamma coactivator-1alpha (*Pgc-1a*), deiodinase (*Dio2*), fatty acid transporter (*Slc27a*), and cytochrome c oxidase subunits 7a/8b (*Cox7a*/*Cox8b*) (Tran et al., 2018; Adachi et al., 2022). However, rodent abdominal aortic PVAT (aPVAT) consists of both brown and white adipocytes, expressing typical white fat markers, such as homeobox C8 (*Hoxc8*), neuronatin (*Nnat*), synuclein gamma (*Sncg*), and mesoderm specific transcript (*Mest*), as well as some brown fat markers at a relatively lower level compared to tPVAT (Tran et al., 2018). Small resistant arteries such as mesenteric (mPVAT) are wrapped with white adipocytes characterized by large unilocular lipid droplets and a lower mitochondrial density (da Silva et al., 2021). The mPVAT expresses a high level of adipogenic transcription factor 21 (*Tcf21*), *Hoxc8*, and *Hoxc9* (de Jong et al., 2015). In human, however, most studies have focused on PVAT enveloping coronary arteries (cPVAT), which is a part of epicardial adipose tissue (Chatterjee et al., 2013). Although cPVAT contains a large proportion of white adipocytes with unilocular and small lipid droplets, it exhibits a transcriptional signature of beige adipose tissue with a high level of expression of thermogenesis-associated genes (Chatterjee et al., 2013; Gaborit et al., 2015).

These PVAT phenotypes can undergo transformation, at least in rodents, in response to certain stimuli (Police et al., 2009; Bailey-Downs et al., 2013). As an example, in high-fat diet-induced obesity, adipocyte in mouse mPVAT, aPVAT, and tPVAT underwent marked hypertrophy in size (Police et al., 2009; da Silva et al., 2021). As mouse aging, these three PVATs from rats also encountered white-like changes, such as increased white adipocytes in number and upregulated levels of white fat markers (Padilla et al., 2013; Garcia-Carrizo et al., 2017). The whitening process during obesity and aging not only stimulates local oxidative stress and inflammation in aPVAT or mPVAT, but also weakens the anti-contractile activity of tPVAT (da Silva et al., 2021; Padilla et al., 2013; Garcia-Carrizo et al., 2017). On the other hand, mouse PVAT undergoes a transition from white to brown in response to cold exposure or β3-adrenergic receptor agonists (Bussey et al., 2018; Mestres-Arenas et al., 2021). In these situations, mPVAT and aPVAT show brown-like properties with increased brown adipocytes and decreased lipid incorporation. This re-browning process is similarly witnessed in tPVAT (Mestres-Arenas et al., 2021). Besides, a long-term aerobic exercise promoted browning of mPVAT and tPVAT, particularly restoring obesity-induced mPVAT dysfunction (Liao et al., 2021).

### 2.2 Heterogenous origin of PVAT depots

Adipocytes contribute to more than 80% of adipose tissue volume (Shen et al., 2003). Mature adipocytes derived from adipocyte precursors (also referred to as adipocyte progenitors or preadipocytes) through *de novo* differentiation (Berry and Rodeheffer, 2013). Using mouse models, scientists have made efforts to reveal the developmental origins of adipocytes in PVAT, especially tPVAT (Fu et al., 2019; Ye et al., 2019). The tPVAT has histological features and transcriptional profiles similar to interscapular brown adipose tissue that primarily originate from myogenic factor 5 (*Myf5*)-expressing progenitors (Sanchez-Gurmaches and Guertin, 2014). However, Ye et al. (2019) found that only 10%–30% of adipocytes in tPVAT derived from *My5*-expressing progenitors. There are three strip-shaped adipose tissues, including anterior tPVAT, left lateral tPVAT, and right lateral tPVAT, constituting tPVAT depot. Ye’s study also demonstrated that 89% of anterior tPVAT (13% *Myf5* ^*+*^) and 62% of left lateral tPVAT (24% *Myf5* ^*+*^) were smooth muscle protein 22 alpha (*SM22α*) positive, and the left lateral tPVAT observed on postnatal day 2 grew up faster than the anterior tPVAT shown on postnatal day 3 (Ye et al., 2019). Subsequently, the authors further discovered that periaortic arch adipose tissue, a type of brown adipose tissue surrounding ascending aorta, originated from at least three types of precursors, including *Sm22a* ^*+*^ neural crest cells, *Myf5* ^*+*^ progenitors, and the other unknown origin (Fu et al., 2019). The periaortic arch adipose tissue was visible on postnatal day 2 and fully developed 1 month after birth, 82% of *Sm22a* ^*+*^ neural crest cells and 11% of *Myf5* ^*+*^ progenitors contributing to the formation of adipocytes (Fu et al., 2019). However, neural crest cells did not contribute to the formation of tPVAT (Ye et al., 2019). More recently, Angueira et al. (2021) unambiguously demonstrated that the thoracic aorta in an 18-day-old embryo was mainly surrounded by fibroblasts expressing platelet derived growth factor receptor alpha (*Pdgfra*). During the early postnatal day 1 to the day 3, there are two precursors involved in PVAT formation, including fibroblastic progenitor that expressed lymphocyte antigen 6 family member a (*Ly6a*) and peptidase inhibitor 16 (*Pi16*), as well as peroxisome proliferator-activated receptor gamma (*Pparg*) positive fibroblastic preadipocytes (*Pdgfra* ^*+*^ *Ly6a* ^*+*^) (Angueira et al., 2021). Interestingly, in adult mice, a new population of smooth muscle cells that expressed myosin heavy chain 11 (*Myh11*), actin alpha (*Acta*), and transgelin 2 (*Tagln2*), also exhibited adipogenic differentiation capacity (Angueira et al., 2021).

Very few studies have focused on the development of other PVATs. Chang et al. (2012) suggested that aPVAT, tPVAT, and periaortic arch adipose tissue, may share the same *Sm22a*-expressing precursors as vascular smooth muscle cells (VSMCs), and deficient of *Pparg* in smooth muscle cells resulted in the deprivation of PVAT in mouse. In contrast, mPVAT shared a similar transcriptional profile with visceral adipose tissue and had the same wilms tumor protein (*Wt1*)-positive progenitor (Chau et al., 2014). Similarly, epicardial adipocyte also originated from *Wt1*-expressing mesenchymal cells, which was transformed from *Tbx18*-expressing epicardial progenitor and further differentiated to mature adipocytes after activation of *Pparg* expression (Chau et al., 2014; Yamaguchi et al., 2015). Collectively, the developmental origins of PVAT adipocytes are complicated. Further studies are essential to clarify the heterogenous origins of PVATs, which lead to the phenotypical and secretory differences among PVAT depots.

## 3 Multifaced cross-talks between PVAT depots and neighbors

**FIGURE 1:**
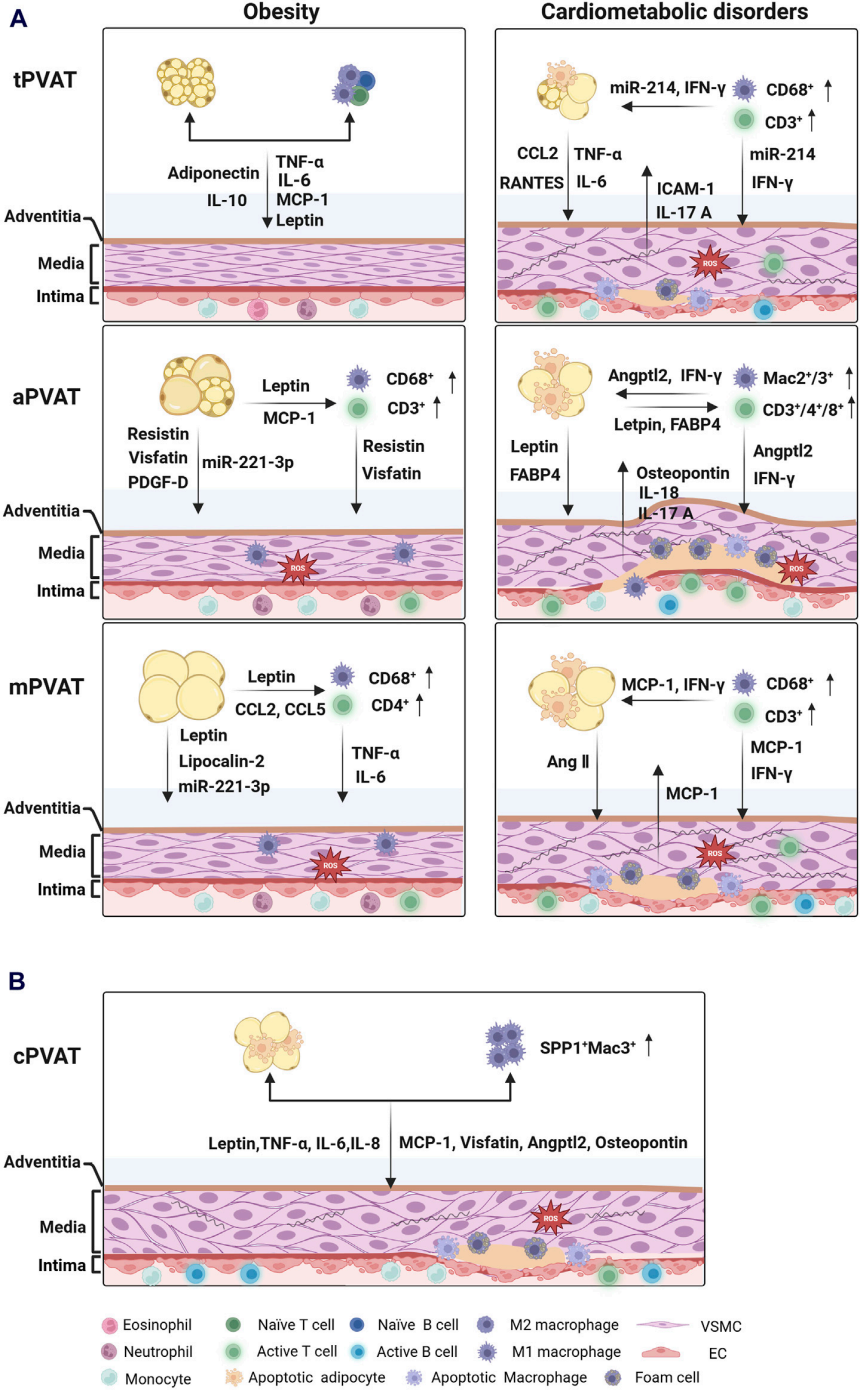
Intercellular communications between PVAT depots and blood vessels under pathological conditions. **(A)** Cross-talks between tPVAT, aPVAT, mPVAT and their nearby blood vessels in rodents. tPVAT exerts great resistance to obesity without obvious infiltration of immune cells. In contrast, aPVAT and mPVAT exhibit hypertrophic and dysfunctional adipocytes, local inflammation, and heightened oxidative stress in the development of cardiometabolic disorders. **(B)** Cross-talks between human cPVAT and the coronary artery in coronary artery diseases. Inflamed cPVAT contributes to the progression of atherosclerosis in the coronary artery. (created with BioRender.com). Abbreviations: Angptl2, angiopoietin-like protein 2; Ang Ⅱ, Angiotensin Ⅱ; FABP4, fatty acid-binding protein 4; "ICAM-1, Intercellular adhesion molecule 1; IFN-γ, Interferon-γ; IL, Interleukin; MCP-1 (CCL2), Monocyte chemoattractant protein-1 (C-C motif chemokine ligand 2); PDGF-D, platelet derived growth factor-D; RANTES (CCL5), Regulated upon activation, normal T cell expressed and presumably secreted (C-C motif chemokine ligand 5); TNF-α, Tumor necrosis factor-α; PVAT, perivascular adipose tissue; tPVAT, thoracic aortic PVAT; aPVAT, abdominal aortic PVAT; mPVAT, mesenteric PVAT; cPVAT, coronary PVAT; VSMC, vascular smooth muscle cell; EC, endothelial cell.

PVAT depots secret a family of adipokines that possess protective or detrimental effects on vascular structure and function. In lean subjects, either human or rodent, PVAT demonstrates a remarkable ability to shield adjacent vessels from inflammation and promote vasorelaxation (Kassan et al., 2011; Adachi et al., 2022). However, in obese individuals, the vasoprotection of PVAT is compromised, accompanied by a plethora of pro-inflammatory and pro-contracting mediators released in PVAT microenvironment (Police et al., 2009). Dysfunctional PVAT triggers vascular oxidative stress and inflammation, which is associated with an imbalance of immunity (Lee et al., 2022). Physiologically, infiltrating immune cells constituted a small fraction, approximately 2%, of stromal vascular fraction cells in mouse PVAT (Mikolajczyk et al., 2016). Nevertheless, in obesity-induced hypertension, this proportion reached 7%–10%, and in atherosclerotic mouse, it surged to 10%–20% (Skiba et al., 2017; Kumar et al., 2021). Macrophages and T cells are two predominant immune cell types in PVAT depots, and the phenotypic changes of those cells are involved in the progression of obesity and cardiovascular diseases (Guzik et al., 2007; Kumar et al., 2021). Distinct PVAT depots indeed generate diverse types and quantities of adipokines, cytokines, and chemokines (Table 1), which serve as messengers mediating multifaced intercellular communications among adipocytes, immune cells, VSMCs, and endothelial cells in PVAT microenvironment (Figure 1).

**TABLE 1:** Comparison of secretome within PVATs under physiological and pathological conditions. These secreted mediators are generally considered as biomarkers of risk factors for the corresponding pathological conditions, such as obesity, type 2 diabetes, or cardiometabolic disorders.

PVATs	Species	Physiology	Pathology	
			Obesity or type 2 diabetes	Cardiometabolic disorders
tPVAT	Rodents	NRG4 (Adachi et al., 2022) COMP (Huang et al., 2023) FGF21 (Mestres-Arenas et al., 2021) Adiponectin, IL-10, Sirtuin1 (Schütz et al., 2019) IL-4 (Liu et al., 2017) BMP4 (Mu et al., 2021) NO (Xia et al., 2016) H_2_O_2_ (Gao et al., 2007) PAME (Lee et al., 2011) H_2_S (Mitidieri et al., 2022) CTRP9 (Han et al., 2018) Ang 1–7 (Lee et al., 2009) Leptin (Lembo et al., 2000; Gálvez-Prieto et al., 2012) Adenosine (Kong et al., 2018)	Leptin, CCL2 (Police et al., 2009) TNF-α, IL-6 (Chang et al., 2018)	RANTES, IFN- γ (Mikolajczyk et al., 2016) TNF-α, IL-6, CCL5, CXCL10, ICAM-1 (Skiba et al., 2017) Ang II (Lee et al., 2011) IL-1β (Mitidieri et al., 2022); miR-214 (Nosalski et al., 2020); CCL2 (Nakladal et al., 2022) IL-17A (Smith et al., 2010)
aPVAT	Human	Adiponectin, Apelin (Kostopoulos et al., 2014)		Leptin, FABP4, IL-18 (Liu et al., 2020) IL-8, MCP-1 (Henrichot et al., 2005)
	Rodents	Adiponectin, IL-10 (Padilla et al., 2013) FGF21, NRG4 (Mestres-Arenas et al., 2021) NO (Victorio et al., 2016)	Leptin, MCP-1 (Police et al., 2009); miR-221-3p, IL-1β, CCL3 (Li et al., 2019); TNF-α, IL-6 (Li et al., 2017) Resistin, Visfatin (Park et al., 2014) PGDF-D (Zhang et al., 2018)	RANTES, IFN- γ (Mikolajczyk et al., 2016) IL-17A (Smith et al., 2010) Resistin, Visfatin (Park et al., 2014) PGDF-D (Zhang et al., 2018) FABP4, IL-18 (Liu et al., 2020) Angptl2 (Tazume et al., 2012)
mPVAT	Rodents	IL-10 (Kassan et al., 2011) Adiponectin (Sena et al., 2017) NO (Gil-Ortega et al., 2010) H_2_S (Schleifenbaum et al., 2010) PAME (Chang et al., 2020)	miR-221-3p, CCL3 (Li et al., 2019) Leptin, CCL2, CCL5, Lipocalin-2 (Sena et al., 2017) TNF-α, IL-6 (Terra et al., 2009)	IL-1β (Lohmann et al., 2009) Ang II, Prostacyclin, Thromboxane A_2_ (Mendizábal et al., 2013) MCP-1, IFN- γ (Mian et al., 2016)
cPVAT	Human	Omentin (Gaborit et al., 2015) Adiponectin, IL-10 (Gruzdeva et al., 2019) Apelin (Kostopoulos et al., 2014) AMAC-1 (Hirata et al., 2011)		MCP-1, IL-8 (Chatterjee et al., 2009) Leptin, IL-6, TNF-α, Visfatin (Cheng et al., 2008) Angptl2 (Tian et al., 2013) Osteopontin (Fu et al., 2023)

Abbreviations: AMAC-1, Alternative macrophage activation-associated CC-chemokine-1; Angptl2, angiopoietin-like protein 2; Ang, Angiotensin; BMP4, Bone morphogenetic protein 4; COMP, cartilage oligomeric matrix protein; CTRP9, C1q/tumor necrosis factor-related protein 9; CXCL10, C-X-C motif chemokine ligand 10; FABP4, fatty acid-binding protein 4; FGF21, Fibroblast growth factor 21; ICAM-1, Intercellular adhesion molecule 1; IFN-γ, interferon amma; IL, interleukin; H_2_O_2_, hydrogen peroxide; H_2_S, hydrogen sulphide; MCP-1 (CCL2), Monocyte chemoattractant protein-1 (C-C motif chemokine ligand 2); MIP-1α (CCL3), macrophage inflammatory protein-1α (C-C motif chemokine ligand 3); NO, nitric oxide; NRG4, Neuregulin 4; PAME, palmitic acid methyl ester; PDGF-D, platelet derived growth factor-D; RANTES (CCL5), Regulated upon activation, normal T cell expressed and presumably secreted (C-C motif chemokine ligand 5); TNF-α, Tumor necrosis factor-α; PVAT, perivascular adipose tissue; tPVAT, thoracic aortic PVAT; aPVAT, abdominal aortic PVAT; mPVAT, mesenteric PVAT; cPVAT, coronary PVAT.

### 3.1 Cross-talks between tPVAT and thoracic aorta

Thoracic perivascular adipose tissue (tPVAT) resembles brown adipose tissue, with a higher tendency of anti-inflammatory and anti-contractile than other PVAT depots. Compared to abdominal aorta, thoracic aorta has a lower incidence of atherosclerotic plaque formation, and this resilience is related to the distinct secretory profile of tPVAT. Most studies regarding the role of tPVAT in regulating vascular function are based on rodent models. For instance, mouse tPVAT releases a plethora of anti-inflammatory adipokines, such as adiponectin (Schütz et al., 2019), interleukin (IL)-10 (Schütz et al., 2019), IL-4 (Liu et al., 2017), and neuregulin 4 (NRG4) (Adachi et al., 2022), which can dampen local inflammation and oxidative stress through inhibiting macrophage polarization or regulating vascular remodeling. NRG4 is a new anti-inflammatory adipokine involved in communication between brown fat and arteries, and NRG4 deficiency has been shown to aggravate thoracic atherosclerosis (Shi et al., 2022). Bone morphogenetic protein 4 (BMP4) has been reported as a browning regulator of tPVAT protecting the thoracic artery against atherosclerosis (Mu et al., 2021). Most recently, tPVAT-derived cartilage oligomeric matrix protein (COMP) has been reported to suppress VSMC apoptosis and to attenuate aortic aneurysm formation in mice (Huang et al., 2023). Besides, tPVAT is a source of abundant vasodilators, including adiponectin, nitric oxide (NO) (Xia et al., 2016), hydrogen peroxide (H_2_O_2_) (Gao et al., 2007), palmitic acid methyl ester (PAME) (Lee et al., 2011), hydrogen sulphide (H_2_S) (Mitidieri et al., 2022), C1q/tumor necrosis factor-related protein 9 (CTRP9) (Han et al., 2018), and angiotensin (Ang) 1–7 (Lee et al., 2009). Additionally, tPVAT-derived leptin might also be a vasodilator of thoracic aorta via enhancing NO production in endothelium (Lembo et al., 2000; Gálvez-Prieto et al., 2012).

The tPVAT exhibited great vasoprotective functions in response to cold exposure and obesity development. A short-term (4 weeks) high-fat diet feeding or cold exposure activated tPVAT browning, accompanied by upregulation of batokines (a collective term for brown adipose tissue-derived active molecules), including bone morphogenetic protein-8b (BMP8B), fibroblast growth factor-21 (FGF21), and kininogen-2 (KNG2) (Mestres-Arenas et al., 2021). Lee et al. (2022) discovered that 12 weeks of high-fat diet feeding did not lead to tPVAT inflammation, including immune cell infiltration and secretion of pro-inflammatory adipokines. Contrarily, in obese rats fed high-fat diet for 16 weeks, higher inducible nitric oxidase (iNOS) expression and reactive oxygen species (ROS) production were witnessed in tPVAT, resulting decreased NO generation and vascular relaxation in adjacent blood vessels (Sousa et al., 2019). Further, 48-week of high-fat diet feeding remarkably enhanced the pulse wave velocity and arterial stiffness in mouse, along with higher level of tumor necrosis factor (TNF)-α, IL-6 and monocyte chemoattractant protein-1 (MCP-1) in tPVAT (Chang et al., 2018). The expression of mitoNEET, an iron-containing protein in the outer mitochondrial membrane, was negatively associated with tPVAT hypertrophy during obesity (Chang et al., 2018). MitoNEET could accelerate lipid uptake and glucose utilization, and a reduction of mitoNEET expression led to oxidative stress and insulin resistance in adipocytes of obese mice (Kusminski et al., 2012). Transplantation of tPVAT from obese mouse promoted wire injury-induced neointimal formation in carotid artery in low-density lipoprotein receptor (*Ldlr*) knockout recipients (Manka et al., 2014). However, this damage was not existing while C-C motif chemokine ligand 2 (*Ccl2*) was knockout in donor mouse, suggesting the proinflammatory role of CCL2 (also known as MCP-1) in development of obesity and vascular pathology. An inflamed tPVAT and stiffening thoracic aorta were observed in leptin-deficient (*ob/ob*) mice, and this impairment was improved through pioglitazone-mediated PPARγ activation (Chen et al., 2021). Moreover, aging promoted obesity-induced tPVAT dysfunction and vascular inflammation in mouse, accompanied by downregulation of NADPH oxidase 1 (*Nox1*) and upregulation of NLR family pyrin domain containing 3 (*Nlrp3*) (Bailey-Downs et al., 2013; Fleenor et al., 2014).

Similarly, tPVAT of 8-week-old spontaneously hypertensive rats (SHR) exhibited an increased browning markers and an equal vasodilation as compared to 4-week-old normotensive rats (Kong et al., 2018). On the contrary, these functions were markedly diminished in tPVAT from 16-week-old SHR, along with reduced plasma adenosine level, increased blood pressure, and endothelial dysfunction (Kong et al., 2018). Adenosine has been participated in activating thermogenesis and increasing lipolysis in both human and murine brown adipose tissue through binding to adenosine A_2A_ receptor (Gnad et al., 2014). Besides, the tPVAT of hypertensive SHR (18–20 weeks old) released a high level of angiotensin (Ang) II, which constricted rat aorta and increased blood pressure (Lee et al., 2011). After 7 days of Ang II infusion, mice aPVAT exhibited elevated inflammatory responses, while tPVAT counterbalanced Ang II-induced inflammation by upregulating brown adipocytes markers (Wang et al., 2022). These data demonstrate that the activated thermogenesis was like a forewarning, and the anti-constriction ability of tPVAT is gradually weaken with the development of hypertension. However, 14 days of Ang II administration increased the expression of RANTES (regulated on activation, normal T cell expressed and secreted, also known as CCL5) in tPVAT of mice (Mikolajczyk et al., 2016). Knockdown of *Ccl5* reduced the infiltration of CD68^+^ and CD3^+^CD4^−^CD8^−^ T cells within tPVAT of mice, accompanied by a decreased production of interferon gamma (IFN- γ). In addition, infiltrating CD3^+^ T cells in tPVAT highly expressed miR-214, which enhanced tPVAT inflammation, worsened aortic endothelial dysfunction, and increased blood pressure in Ang II-loaded mice (Nosalski et al., 2020).

In initial stage of atherosclerosis, although tPVAT exhibited increased CD68^+^ macrophage infiltration and iNOS expression, the mRNA level of *Ccl2*, *Il6* and *Adipoq* remained unchanged in lipoprotein E (*ApoE* ^*−/−*^) knockout rats (Nakladal et al., 2022). This moderate inflammatory response in tPVAT yet boosted acetylcholine-mediated endothelium relaxation in an NO-dependent manner (Nakladal et al., 2022). The protective function of tPVAT on endothelium was also observed in *Ldlr* ^*−/−*^ mice (Baltieri et al., 2018). Nevertheless, tPVAT was enriched in CD68^+^ and CD3^+^ T cells during atherogenic plaque formation, while the thoracic aorta displayed collagen deposition and infiltration of CD4^+^ T helper 17 cells. This created a detrimental interaction between PVAT and blood vessels (Smith et al., 2010; Skiba et al., 2017). Taken together, tPVAT shows superior resistance in the early stage of obesity and cardiometabolic diseases, but excessive accumulation of macrophages and T cells disrupts its functionality (Figure 1A).

### 3.2 Cross-talks between aPVAT and abdominal aorta

The perivascular adipose tissue surrounding abdominal aorta (aPVAT) contains both brown and white adipose tissues. Based on its structural features, aPVAT exhibits thermogenesis similar to tPVAT in response to cold exposure and shows resistance to a short-term high-fat diet feeding (Mestres-Arenas et al., 2021). Moreover, aPVAT can produce NO, but it exhibits a dramatic reduction in eNOS expression compared to tPVAT in the presence of phenylephrine (Victorio et al., 2016). A recent study demonstrated that all-trans-retinoic acid increased adiponectin expression and secretion in aPVAT, augmented NO production in the abdominal aorta, and attenuated atherosclerotic plaque formation in *ApoE* ^*−/−*^ mice (Kalisz et al., 2021). These findings imply the important role of aPVAT-derived adiponectin in regulating vascular function of the abdominal aorta.

However, aPVAT expresses higher levels of inflammatory adipokines and immune cell markers, but lower levels of adiponectin and IL-10 than tPVAT under basal condition (Padilla et al., 2013). These might be the reasons why abdominal aorta is more vulnerable to obesity, atherosclerosis, and aneurysm than thoracic aorta. Abdominal aortic aneurysm (AAA) causes 1% of deaths in men over 65 years old, and the mortality rate rapidly increases with age (Howard et al., 2015). Abdominal adiposity, featured by an increased waist circumference, is also recognized as a main risk factor of AAA for patients with obesity (Stackelberg et al., 2013). Here, we discuss the association between aPVAT dysfunction and the pathogenesis of atherosclerosis and AAA, with a focus on the impact of obesity on aPVAT inflammation.

Obesity promoted hypertrophy and inflammation in aPVAT. The aPVAT from obese rodents expressed high levels of pro-inflammatory genes such as *Tnfa*, *Il6*, *Il1b*, *Ccl2*, and *Ccl3* compared to those from lean counterparts (Li et al., 2017; Li et al., 2019). In both diet-induced obese and diabetic mouse (*ob/ob*) models, aPVAT exhibited increased CD68^+^ macrophages infiltration and a high expression of resistin and visfatin (Park et al., 2014). The authors also uncovered that macrophages were a main source of resistin and visfatin in aPVAT, and these two adipokines promoted osteopontin expression in VSMCs through activation protein 1 (AP-1)-mediated signaling (Park et al., 2014). Additionally, increased osteopontin expression has been implicated in atherosclerotic plaque formation and AAA progression (Bruemmer et al., 2003). Subsequently, Police et al. (2009) noted that in obesity, aPVAT was markedly expanded and enriched with MCP-1 and leptin. At the same time, aPVAT was infiltrated by a substantial accumulation of CD68^+^ macrophages, contributing to the development of Ang II-induced AAA during obesity (Police et al., 2009). Recently, platelet derived growth factor-D (PDGF-D) was reported to be highly expressed in aPVAT of obese mice, and this enhanced expression accelerated adventitial fibrosis and inflammation during obesity-related AAA formation (Zhang et al., 2018). Here, adipocyte-derived PDGF-D promoted adventitia thickening and secretion of inflammatory factors through TGF-β/Smad signaling (Zhang et al., 2018). In Ang Ⅱ-induced hypertension, aPVAT contained abundant CD68^+^ macrophages and CD3^+^ T cells, as along with increased expression of RANTES and IFN- γ (Mikolajczyk et al., 2016).

On the other way, aPVAT-residing adipocytes could release leptin and fatty acid-binding protein 4 (FABP4) to promote IL-18 targeting to macrophages, VSMCs, and ECs in mouse with AAA (Liu et al., 2020). In this case, increased expression levels of IL-18 and IL-18 receptor subsequently recruited Mac2^+^/Mac3^+^ macrophages and CD4^+^/CD8^+^/CD3^+^ T cells infiltration and aggravated inflammation in aortic wall (Liu et al., 2020). The infiltrated Mac2^+^ macrophages had been demonstrated to promote the enlargement of CaCl_2_-induced mouse AAA through production of angiopoietin-like protein 2 (Angptl2) (Tazume et al., 2012). Angptl2 was primarily identified as an adipocyte-derived inflammatory mediators that contributes to adipose tissue inflammation and systemic insulin resistance in obese mice (Tabata et al., 2009). In human, aPVAT from patients undergoing abdominal plastic surgery also exhibited an accumulation of CD68^+^ macrophages and CD3^+^ T cells, as well as a high expression of IL-8 and MCP-1 (Henrichot et al., 2005). Recently, an elevated AP-1 genes family, including *Fos*, *Fosb*, *Atf3*, *Jun* and *Junb*, was observed in aPVAT from patients with AAA. The expression of these genes was strongly correlated with activated mast cell and T helper cells in aPVAT from AAA patients (Ding et al., 2022). In summary, obesity-induced aPVAT inflammation and vascular remodeling triggers the initiation and development of AAA (Figure 1A).

### 3.3 Cross-talks between mPVAT and mesenteric artery

Mesenteric artery consists of many small peripheral resistance arteries that start from the abdominal aorta and extend to digestive organ intestine. The mesenteric adipose tissue (mPVAT) helps balance arterial lipid homeostasis and provides energy for nearby intestines and arteries. Besides, mPVAT can regulate the systemic blood pressure through releasing adipokines into mesenteric artery. In physiological setting, mouse mPVAT secrets vasodilators, such as adiponectin (Sena et al., 2017), NO (Gil-Ortega et al., 2010), H_2_S (Schleifenbaum et al., 2010), and PAME (Chang et al., 2020), to modulate vascular tone through activation of potassium channels in VSMCs. However, mPVAT tends to be inflamed in the context of obesity, hypertension, and atherosclerosis. The mPVAT from obese rat exhibited high concentrations of pro-inflammatory substances, such as leptin, chemokine ligands (CCL2, CCL5), and lipocalin-2 (Sena et al., 2017). Meanwhile, dephosphorylation of endothelial NO synthase (eNOS) led to increased ROS production and reduced NO bioavailability, indicating impaired endothelial function in mesenteric arteries of obese rat (Sena et al., 2017). Our previous study indicated that mPVAT from obese moue was able to release extracellular vesicles containing miR-221-3p, which promoted VSMCs phenotype switching and migration (Li et al., 2019). Interestingly, cross-talks between mPVAT and mesentery artery demonstrate sexual dimorphism in response to die-induced obesity. For example, the anti-contractile effect of mPVAT on mesenteric artery of female mice was impaired by 3-month of high-fat plus high-glucose feeding, while the mPVAT dysfunction in males was observed until 5 months (Victorio et al., 2021). This indicated that mPVAT of female animals is more vulnerable to obesity than that of males’ mPVAT (Victorio et al., 2021). The mPVAT-derived Ang II and prostaglandins, including prostacyclin and thromboxane A_2_, blocked vascular response to acetylcholine and promoted endothelial dysfunction in mesenteric artery of rats with metabolic syndrome (Mendizábal et al., 2013).

On the other hand, pro-inflammatory immune cell infiltration is largely involved in mPVAT dysfunction. For example, CD68^+^ macrophages and CD4^+^ T cells were rich in mPVAT from obese mice during progression of hypertension, which was linked to impaired mPVAT-mediated vasodilation (Sena et al., 2017; Kumar et al., 2021). Besides, Mian et al. (2016) demonstrated that Ang II stimulated a great CD3^+^ T cell infiltration in both mPVAT and mesenteric artery, along with an increased MCP-1 expression and IFN- γ production. They found that FOXP3^+^CD4^+^CD25^+^ T cells, a set of T-regulator cells (Tregs), were required for mesenteric artery and neighboring adipose tissue to fight against Ang II-induced inflammatory changes (Mian et al., 2016). Another study reported that CD4^+^CD25^+^ Tregs promoted diastolic blood pressure and mesenteric artery relaxation in hypertensive mice (Kassan et al., 2011). These Tregs inhibited NADPH oxidase activity and increased eNOS phosphorylation through secretion of IL-10 and transforming growth factor beta (TGF-β), significantly reducing endothelial dysfunction and oxidative stress in mesenteric artery (Kassan et al., 2011). However, in atherosclerotic mice, a constitutive CD68^+^ macrophages and CD3^+^ T cells homed into mPVAT and mesenteric arterial wall (Lohmann et al., 2009). Collectively, the phenotypic changes of immune cells impact metabolism and intercellular communication in mPVAT microenvironment (Figure 1A).

### 3.4 Cross-talks between cPVAT and coronary artery

Human coronary perivascular adipose tissue (cPVAT) is an important part of epicardial adipose tissue that particularly surrounds coronary arteries and heart muscle. The cPVAT is responsible for regulating blood flow in coronary artery and provides free fatty acid as an energy source for myocardium (Heymes et al., 2003). Unlike classic white adipose tissue, cPVAT is composed of white adipocyte with unilocular and small lipid droplets, resulting in a distinct metabolism. Omentin, an adipokine highly expressed in cPVAT, modulates the inflammatory response and coronary dilation during the development of coronary artery disease (CAD) (Cheng et al., 2008; Gaborit et al., 2015). Under ischemic condition, cPVAT-derived omentin promoted blood flow recovery and revascularization via activation of endothelial cell function (Maruyama et al., 2012). Adrenomedullin is an antioxidant peptide originated from preadipocyte or CD68^+^ macrophages in cPVAT, which plays a cytoprotective role in chronic CAD (Silaghi et al., 2007). Adiponectin and apelin have also been considered as potential anti-atherogenic adipokines in human cPVAT (Kostopoulos et al., 2014; Gruzdeva et al., 2019). Adiponectin can bind to a glycosylphosphatidylinositol-anchored protein T-cadherin in VSMCs and ECs, subsequently controlling vasodilation of the adjacent vessel (Kostopoulos et al., 2014).

However, in patients with obesity or type 2 diabetes, cPVAT loses the capacity to balance excessive free fatty acids in adjacent myocardium and coronary vascular bed, which promotes accumulation of myocardial triglyceride, disturbs contractile reactivity of heart, and even results in lipotoxic cardiomyopathy (Hammer et al., 2008; Viljanen et al., 2009). Additionally, a quite lower expression of adiponectin and apelin was observed in aged population and patients with hypertension, which could be associated with the pathogenesis of CAD (Teijeira-Fernandez et al., 2008; Chatterjee et al., 2009). Many clinical studies have demonstrated that cPVAT enlargement and inflammation are related to coronary artery calcification and plaque vulnerability in the atherogenesis (Nomura et al., 2020). In patients with CAD, cPVAT exhibited heightened concentrations of pro-inflammatory adipokines and cytokines, such as leptin, TNF-α, IL-6, MCP-1, visfatin, and Angptl2 (Cheng et al., 2008; Tian et al., 2013). After receiving coronary artery bypass graft surgery, patients showed enhanced infiltration of CD68^+^ macrophages in their cPVATs, where the number of both CD11c^+^ macrophages (M1) and CD206^+^ macrophages (M2) was increased, and the polarization of M1 macrophages was evidenced by an augmented ratio of CD11c/CD206 (Gruzdeva et al., 2019). Consistently, the mRNA expression of cytokines IL-10 and alternative macrophage activation-associated chemokine-1 (AMAC-1) secreted by M2 macrophage was also upregulated, but the expression was negatively correlated with the polarization of M1/M2 macrophages (Hirata et al., 2011; Gruzdeva et al., 2019). Subsequently, macrophage accumulation and polarization in cPVAT further induced inflammatory flux into vascular adventitia, interfering with plaque stability and neovascularization. Additionally, a high level of secretory type II phospholipase A2 (sPLA2) was found in human atherosclerosis lesion, and prospective evidence has demonstrated that this phospholipase serves as an independent risk factor of CAD (Ivandic et al., 1999). The sPLA2 is an enzyme hydrolyzing phospholipids to produce lysophospholipids and dominantly expressed in macrophage rather than adipocytes in cPVAT from patients with CAD (Dutour et al., 2010). Most recently, a single-cell RNA sequencing study revealed that Mac3^+^ macrophages expressing *SPP1* (secreted phosphoprotein 1) were identified in cPVAT of patients in the end stage of CAD (Fu et al., 2023). This macrophage subpopulation secreted osteopontin and influenced the proliferation and migration of fibroadipogenic progenitor, thus promoting fibrosis in cPVAT and increasing coronary stenosis grade (Fu et al., 2023). These findings indicate that cPVAT inflammation is a pathological link between obesity and increased risk of CAD (Figure 1B).

In addition, Ahmad et al. (2017) found that the anti-contractile effect of cPVAT on pig’s coronary arteries exhibited sexual differences in response to U46619 (9,11-Dideoxy-11α, 9α-epoxymethano-prostaglandin F_2α_). The cPVAT from females, but not males, regulated vasodilatory function of coronary artery when exposed to U46619, and this effect was not associated with adiponectin expression and secretion (Ahmad et al., 2017). This indicates that cPVAT from different genders possesses different intracellular signaling pathways in the regulation of coronary artery tone. However, whether this sexual difference is conserved in humans is not clear. In comparison to lean counterparts, pigs with metabolic syndrome exhibited a larger cPVAT in volume and coronary endothelial dysfunction (Payne et al., 2010). In this context, the cPVAT-derived leptin was suggested as a contributor to the onset of coronary atherogenesis.

## 4 PVAT as clinical biomarkers and therapeutic targets

PVAT, as a special type of adipose tissue, has increasingly been recognized as not only a clinical biomarker but also a target for the treatment of cardiometabolic diseases. Computed tomography (CT) imaging is a well-established method to monitor lipid deposition in human cPVAT and to diagnose coronary artery disease (Antonopoulos et al., 2017; Ueno et al., 2021). The lipid accumulation and adipose tissue expansion of cPVAT can be quantified by the fat attenuation index (FAI) in a CT scan which captures coronary inflammation by mapping spatial changes of perivascular fat attenuation (Antonopoulos et al., 2017). When the blood vessel was inflamed, the FAI of its cPVAT exhibited a gradient from the adventitial layer of coronary artery to the outer layer of cPVAT (Antonopoulos et al., 2017). Recently, Lee et al. (2023) used the CT imaging to evaluate the differences of aPVAT FAI between patients with mild metabolic syndrome and healthy ones. They found that the volume of aPVAT was positively correlated, while the FAI was negatively correlated with metabolic disorders. It is unfortunate that this CT technology is limited in their ability to assess the proportions or detailed structure of mPVAT and tPVAT.

In addition, preclinical strategies such as tPVAT transplantation and β3-adrenergic receptor agonists have been developed. Huang et al. (2023) reported that transportation of tPVAT from health donors improved the vascular function and inhibited the development of abdominal aortic aneurysms in recipients. CL316243, a β3-adrenergic receptor agonist, was shown to represses vascular remodeling and inflammation through the beiging of PVAT around mouse femoral artery (Adachi et al., 2022). However, some evidences have demonstrated that drugs (e.g., mirabegron) or cold-induced browning exacerbates the progression of atherosclerotic lesion (Dong et al., 2013; Sui et al., 2019). In these two studies, levels of plasma low-density lipoprotein cholesterol and total cholesterol were increased, while the triglyceride concentration decreased in mice. Cardiovascular diseases are associated with a cluster of metabolic disorders, including non-alcoholic fatty liver, obesity, and insulin resistance (Semmler et al., 2023). These findings indicate that cold exposure and mirabegron induce increased lipolysis of adipose tissue throughout the body, but they might disrupt the balance of glucose and lipid metabolism in other organs, ultimately resulting in lipid deposition in circulation. Therefore, further investigation of depot-specific therapeutic strategies is needed for the prevention and treatment of cardiometabolic disorders in the long run.

## 5 Conclusion and perspectives

The structural, phenotypic, and original differences among PVAT depots are closely associated with their anatomical location. These regional differences result in spatially distinct secretome, a cluster of mediators which mediate multifaceted interactions within and beyond PVAT. The bi-directional cross-talk between PVAT depots and the vascular wall differs in response to pathological changes (Figure 1). Under physiological conditions, all PVAT depots exert vasoprotective functions. When the vascular wall is overstressed, PVAT depots secrete protective adipokines in a paracrine manner. However, over-hypertrophic PVAT depots fail to maintain vascular tone and release detrimental adipokines, inducing vascular inflammation. Compared to mPVAT and aPVAT, tPVAT exhibits greater resistance to vascular oxidative stress in the early stages of obesity and vasculopathy development (Figure 1A). In fact, aPVAT shows weak resistance to short-term high-fat diet feeding but becomes progressively extended and inflamed after long-term feeding. The mPVAT from obese rodents not only loses its function but also recruits CD4^+^ T cells, which infiltrate into PVAT and the vascular wall, facilitating ROS production and inflammation. Furthermore, impaired vessels release chemokines that activate macrophages and T cells, contributing to immune cell accumulation, lipid deposition, and foam cells formation during the development of cardiovascular diseases. Regarding human cPVAT, increased generation of pro-inflammatory adipokines and accumulation of SPP1^+^Mac3^+^ macrophages have been observed in coronary artery disease (Figure 1B). Adipokines and immune cell phenotypes vary across the anatomical locations of PVATs. Just as each Nereid added a layer of complexity to the mythological tales of the sea, the heterogeneity of PVAT across vascular beds adds intricacies to our understanding of vasculopathy. Exploring the various attributes and functions of PVAT in different regions will offer valuable insights into the mechanisms underlying the development and progression of vascular diseases. By unraveling the individual “personalities” of PVAT, we may unveil the complex interplays between adipose tissue and the vasculature, ultimately leading to potential therapeutic strategies for treating or preventing cardiometabolic disorders, such as hyperlipidemia, hypertension, atherosclerosis, and aortic aneurysms.

Yet, several aspects need addressing in future investigations. First, while many studies delineate secretory profile of PVAT depots based on their white-like or brown-like features, specific genes/cells governing PVAT fate, structure, and function remain unidentified. Therefore, further research is needed to unravel the origins and metabolism of different PVAT depots. Second, mechanisms underlying cell-to-cell interactions within the PVAT microenvironment and subsequent downstream signaling are still inadequately understood. Most research focuses on adipokines’ roles in vascular regulation, often through PVAT removal or transplantation, making it challenging to isolate individual molecules involved in these processes. Moreover, exploring intercellular communication among adipocytes, immune cells, VSMCs, and ECs is intricate. Third, the impact of immune cells on regulating PVAT microenvironment remains largely unexplored. A deeper understanding of immunological changes in PVATs is required to decipher their roles in the development of obesity and cardiometabolic diseases. Fourth, most studies have focused on rodents’ models rather than clinical samples, and whether cross-talks identified in rodent are conserved in human is not sure. Thus, it is essential to confirm the diagnostic values of adipokines in clinics.

Future investigations should focus on developing novel strategies to identify the sources and features of PVAT-derived mediators. Single-cell approaches are crucial for exploring intercellular communication and identifying distinct cell populations. Additionally, employing sophisticated cell fate mapping techniques and comprehensive omics analyses could help tackle key nodes in the intercellular network. Further investigation of depot-specific therapeutic strategies is also needed for the prevention and treatment of cardiometabolic disorders in the long run.
